# Relationship Between Motor Variability, Accuracy, and Ball Speed in the Tennis Serve

**DOI:** 10.2478/v10078-012-0043-3

**Published:** 2012-07-04

**Authors:** Ruperto Menayo Antúnez, Francisco Javier Moreno Hernández, Juan Pedro Fuentes García, Raúl Reina Vaíllo, Jesús Sebastián Damas Arroyo

**Affiliations:** 1 Faculty of Physical Activity and Sport. San Antonio Catholic University Campus de Los Jerónimos, Guadalupe, Spain.; 2 Centro de Investigación del Deporte (CID). Universidad Miguel Hernández Avda. de la Universidad, Elche, Spain.; 3 Sport Science Faculty. University of Extremadura, Cáceres, Spain.

**Keywords:** variability, tennis serve, performance

## Abstract

The main objective of this study was to analyze the motor variability in the performance of the tennis serve and its relationship to performance outcome. Seventeen male tennis players took part in the research, and they performed 20 serves. Linear and non-linear variability during the hand movement was measured by 3D Motion Tracking. Ball speed was recorded with a sports radar gun and the ball bounces were video recorded to calculate accuracy. The results showed a relationship between the amount of variability and its non-linear structure found in performance of movement and the outcome of the serve. The study also found that movement predictability correlates with performance. An increase in the amount of movement variability could affect the tennis serve performance in a negative way by reducing speed and accuracy of the ball.

## Introduction

The tennis serve is the most important stroke in the game of tennis for determining the outcome of the match (Pérez and Nussbaum, 2006). Its influence in the game has been analyzed from different perspectives and with various analytical tools (e.g. biomechanical analysis, motor control and learning, physiology, psychology, notation, statistical analysis). Disciplines such as biomechanics have attempted to describe the kinetic and kinematic parameters of the tennis serve, from movement characteristics (Elliot, 2007; Elliot et al., 2003; 2009). The increase of muscular strength in tennis players, as well as the contribution of several rotation variables in upper extremity joints during the performance (Elliot et al., 2003), have been proposed as the reason for performance achieved in this stroke. However, only a few studies have established a relationship between the serve performance and the result achieved, given that this is a complex task (Menayo, 2010).

Variability appears as a distinguishing feature of someone’s behaviour and even, of his ability to perform a movement in a particular environment. This variability can be found at different levels of movement (Bartlett, 2008). Its presence is caused by interactions among the numerous systems and determining factors taking part in movement production and control. At the same time, they are the immediate result of degrees of freedom associated with movement (Bernstein, 1976; Latash, 2008). Far from being understood as harmful for performance, new researches suggest that when variability appears in motor performance, it may be beneficial for movement organization and performance. In fact, it may represent an index of endurance capacity to determining factors related to this performance. Furthermore, variability appears not only in the motor system, but also in tactical situations raised by the opponent or even under environmental conditions that affect the performance of the stroke and need adaptation by the player (Mendes et al., 2011). From this point of view, it is considered that variability may be a factor to take into account, relative to movement pattern stability. Large amounts of variability may suggest unstable movement patterns, although, if that variability is exploratory of action possibilities, it may generate greater effectiveness in performance (Menayo et al., 2010). For example, advances in technology using lighter and stiffer materials in tennis racket construction have resulted in a reduction of kinematic variability when hitting the ball as these modern materials have reduced vibration upon impact and increased serve speed to well over 200 km/h. In addition, there are researches, mainly about postural stability, which use variability as a possible strength system reference (Nashner and McCollum, 1985) although these researches consider stability to be solely an indicator of resistance to perturbations. In that case, variability would allow flexibility within the neuromotor system to permit learning of new movement patterns through adjusting the appropriate parameters.

Variability in performance may also allow flexibility to select or change to new, previously learned movement patterns, by permitting access to the parameters defining these patterns. Variability may even provide stochastic perturbations (fortuitous) that allow constant sampling of different movement patterns so that the most appropriate pattern can be selected (Newell and Corcos, 1993).

A third interpretation is stated by other authors, who argue that the possible co-existence of variability and stability during movement performance give rise to the release and suspension of degrees of freedom processes (Davids et al., 2008). It is an appropriate resource that could be beneficial for movement control.

The hypothesis of the current research sets up that kinematic variability of the hand during tennis serve performance will be a highly relevant variable in performance of that stroke. In that way, the aim of the current research was to analyze the relationship between the variability measured in the hand holding the racket and the accuracy achieved in this stroke.

## Methods

### Subjects

Seventeen intermediate male tennis players took part in the research (M age = 20.8 ± 2.9 years), body height 173 ± 0.08 cm, and body mass 64.85 ± 9.02 kg. All tennis players had previous experience in national tennis competitions (6.94 ± 2.99 years). They were all right-handed and able to perform first serves. None of the participants played tennis outside the timetable for data collection during the research. All the participants provided informed consent according to the Declaration of Helsinki. The Extremadura University Ethical Committee approved the procedure.

### Measures

Product variables analyzed were stroke accuracy, measured by radial error (Robins et al., 2006), variable error, which represents serve errors made in respect of deviation from the serve target area, and the ball speed. Process variables (Table 1) were measured over the trajectory of the hand holding the racket along the antero-posterior (X), the transverse (Y), and the longitudinal (Z) axes. With respect to non-linear variables, these give information about the structure and characteristics of the variability present in the time series. These time series were derived from the position of the hand holding the racket during its trajectory, from the beginning of the movement until the moment the racket hit the ball.

### Tasks, material and measurements

Each tennis player performed 20 first serves. They were instructed to hit the ball with as much power and accuracy as they could, and to avoid sending the balls into the area known in tennis slang as the “T” (the line intersection which divides both service boxes from their respective service lines). The ball bounce on the tennis court surface was video recorded in every serve (Sony HDR- HC3E). The video camera was set at a height of 3 meters and was positioned at the back of the court.

In order to measure accuracy, a Visual Basic 5.0 application was developed (Menayo, 2010). This facilitated the calculation of real-space Cartesian coordinates for the ball bounces through a digitization process from the video recording of the serves. Non-linear kinematic variables were analyzed by using a software application created with Visual Basic 5.0, from an algorithm for calculating Approximate Entropy (Pincus, 1991).

**Figure 1:**
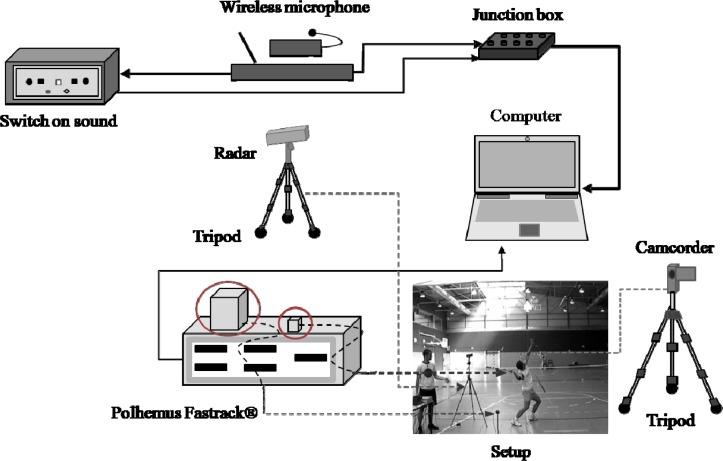
*Automated measurement system*.

To measure ball speed, a radar gun (Sports Radar SR3600) was used. This radar device, which records the speed of moving objects with an accuracy of +/− 1 km/h, was positioned behind the tennis player, facing the direction of the stroke (Figure 1). An electromagnetic motion tracking system Polhemus Fastrak^®^ was used to record and analyze kinematic variables and this was connected to a computer (Toshiba Satellite 1900). This tracking system has 6 Degree-of-Freedom motion tracking sensors, with an accuracy of 0.08 cm for position (X, Y and Z Cartesian space coordinates) and 0.15 degrees for angular orientation (azimuth, elevation, and roll), and records at a frequency of 120 Hz.

The instant of hitting the ball was recorded by a wireless microphone (FoneStar MSH-135) and a sound switch (Lafayette 63040*C), synchronized with Polhemus Fastrak^®^. Figure 1 shows the automated recording system.

Kinematic variables related to position, linear speed and linear acceleration of the hand holding the racket were computed and stored, together with the time series of these variables for each trial made by the tennis players.

### Procedure

The recording process was carried out in an indoor tennis court. The tennis players were called at 30-minute intervals and there was no interaction with other participants. They were given instructions with regard to the task objective, the position to adopt in the service area, the number of trials and the rest periods. Each player performed an individual and specific warm-up of 5 minutes before the execution of the serves. During the warm-up, each tennis player performed 8 serves, with a 10 second pause between serves.

The player adopted the correct position to perform the serves (Figure 1) and a Polhemus Fastrack^®^ receiver was placed on the back of the hand holding the racket. Five serves were performed to check that the recording equipment was working correctly and to allow the tennis player to get used to it. Finally, each tennis player performed a set of 20 serves, with a 20 second pause between trials.

### Statistical analysis

Prior to the statistical analysis, a Kolmogorov-Smirnov test confirmed the normal distribution of the data. For analyzing the relationship between radial error, variable error, accuracy and ball speed in serves, and kinematics and non-linear variables related to performance, a Pearson correlation analysis was carried out. A stepwise regression analysis of the correlated variables was conducted, for the purpose of examining and determining the weight of these variables to predict radial error, and determining the strength of global association, by the multiple correlation coefficient R^2^. Likewise, an adequate effect size through Cohen’s *d* was confirmed.

## Results

Table 2 shows immediate kinematic variables results which have a significant correlation with radial error computed in serves. The significant and positive relationship within the amount of variability at peak linear speed in X, Y, and Z axis, as well as in the time until the linear speed and acceleration peak, means that an increase in variability leads to decreased accuracy (more radial error). The regression analysis shows that the variable defined as the standard deviation of the time to maximum linear speed in X axis (tpvlx_SD) could be the sole predictor variable of 29.7% radial error in the serves (*F*_1,16_ = 6.326; *R*^2^ = .297; ß = .545; *p* = .024; *d* = .06). In this way, variability in the time to reach maximum linear speed of the hand in the antero-posterior axis could be a predictor of accuracy in serves.

Table 3 shows immediate kinematic and non-linear variables which have a significant correlation with variable error computed in serves. The significant and negative relationship within the amount of variability at the beginning of the hand movement in Y axis (poiy_SD) means that a lower dispersion of the serves is related to a higher variability of the hand position. Similar to the results of the radial error, an increase in the time variability until the linear speed peak in X axis (tpvlx_CV) is related to variable error increases. With regard to the non-linear variability, the results show positive and significant relationships between the approximate entropy of the kinematic variables and the variable error (Table 3). This indicates that the tennis players perform more predictable movements regarding position, speed and acceleration of the hand when the error variability is decreased.

Regression analysis applied to these variables determines that the variable defined as the variance ratio of the time to maximum linear speed in X axis coefficient (tpvlx_CV) could be the sole predictor variable of 33.5% variable error in the serves (*F*_1,16_ = 7.550; *R*^2^ = .335; ß = .579; *p* = .015; *d* = .07). Again, variability in the time to reach maximum linear speed of the hand in the antero-posterior axis could be a predictor of accuracy in serves, in this case in the dispersion of same.

Table 4 shows immediate kinematic variables which have a significant correlation with ball speed reached in the serves. The kinematic variability recorded from the position, distance travelled, speed and movement time of the hand holding the racket is related to a decrease in serve speed. Regression analysis applied to these variables determines that the variable defined as the standard deviation of the distance travelled by the hand in Y axis (diy_SD) could be the sole predictor variable of 76.9% ball speed in the serves (*F*_1,16_ = 23.330; *R*^2^ = .769; ß = .675 y 553; *p* < .001; *d* = .09). Therefore, variability in the distance travelled by the hand in the transverse axis could be a predictor of ball speed in serves.

## Discussion

These results show that radial error is related to the amount of kinematic variability in the three space axes (X, Y and Z). When variability of the speed of the hand holding the racket is increased in X axis, accuracy was decreased. This decrease of performance outcome would also arise when variability is increased in maximum linear speed in Y and Z axes, as well as in the time to maximum acceleration of the hand in Z axis. In addition, accuracy is decreased when tennis players increase the amount of variability in maximum linear speed of the hand in Y and Z axes. These results are similar to those previously found with regard to the inverse relationship between movement variability and accuracy in arm movements (Darling and Cooke, 1987). These authors also found a greater amount of variability in short duration movements, as in our study, mainly during the acceleration phase of the movement. The relationship between movement time, speed and acceleration variables, leads us to conclude that an increase in variability results in a decrease in accuracy.

With regard to accuracy, the most influential factor in radial error is the amount of variability in the time to maximum linear speed in X axis. This means that the greater the variability in the time that the tennis player needs to reach maximum hand speed in X axis, the greater the radial error. This may be related to the fact that, with greater movement time, there is more possibility that the neuromotor system introduces stochastic fluctuations that allow constant sampling of different movement patterns so that the most appropriate pattern can be selected (Van Emmerik and van Wegen, 2000).

In this particular case, as concluded by Hamilton and Reinschmidt (1987) and Miller (2002) in their studies in basketball shooting variability, and by Darling and Cooke (1987) and Messier and Kalaska (1999) in their analysis of grasping and reaching movements, we have found an inverse relationship between performance variability, the strength of performance and accuracy in serves. In addition, this variable could provide information about the characteristics present in the task of achieving the player’s desired performance, for strengthening, creating or changing the attractors of performance (Nashner and McCollum, 1985). Therefore, the task suggested in order to generate changes in this variable could assist tennis player’s exploration of the perceptual-motor landscape that surrounds the serve performance (Glazier et al., 2003) and its performance possibilities.

With regard to non-linear variables, a relationship to accuracy in serve has not been found. This result agrees with that found by Pérez and Nussbaum (2006). These researchers concluded that complexity found in time series, analyzed from different positions of body segments (with four-phases of movement analysis), remained relatively constant and stable in time.

Regarding variable error, it is worth pointing out the negative correlation with the amount of variability in the initial position of the hand in Y axis. The increase in the latter indicates the decrease in variable error. Similarly, if the amount of variability at the beginning of the movement of the hand is increased, serve error deviation will decrease. The results confirm the conclusions of a previous javelin throwing study (Best et al., 1995), which determined that performance strength was not related to accuracy. This could lead us to conclude that an increase in the variability of hand position at the beginning of the movement is a transition between states of organization (Kugler and Turvey, 1987) prompted by a search for different hand movement possibilities. Nevertheless, positive correlation of variable error with the amount of variability in maximum linear speed of the hand in X axis presumes that when the hand speed in X axis variability is increased at the ball′s impact, variable error will also be increased. These results are similar to those found in radial error and match with the results of the aforementioned studies about the negative effect of movement variability in accuracy (Darling and Cooke, 1987; Menayo et al., 2010; Hamilton and Reinschmidt, 1997; Messier and Kalaska, 1999). In addition, this variable can best predict variable error. This fact suggests its consideration for the design of tasks related to serve performance.

Regarding non-linear variables related to variable error in serves; it is found that when complexity of hand trajectory in the three axes is increased, variable error is increased as well. The same fact occurs in complexity in speed of the hand in Y and Z axes, as well as in X axis acceleration. The greater the complexity of the kinematics of tennis player’s movement, the greater the error deviation in hand position, speed and acceleration. These results disagree with the prediction made by different authors (Riley and Turvey, 2002) who proposed that at a low level of performance reactions are more predictable and less complex.

Results analysis relating to ball speed show that variability in the distance travelled by the hand in Y and Z axes correlates in a negative way with ball speed. Variability increases in the distance travelled by the hand in Y axis have the greatest negative effect on ball speed. Ball speed also seems to be decreased when range of variability in the final position of the hand in Y and Z axes is increased. In this way, if there is a high variability in the hand position at the instant of hitting the ball, there is a decrease in ball speed. This result could be interpreted in a similar way to the loss of accuracy resulting from a greater variability in performance, as previously mentioned.

From the results concerning ball speed, it would be correct to infer that, similar to the findings regarding accuracy, they appear to show processes of freezing and freeing degrees of freedom (Davids et al., 2008). Such processes would appear to investigate the sampling search between different variables in order to increase or maintain ball speed. More research is necessary to understand the effect of movement variability on performance, with more participants and with different levels of experience.

## Conclusion

On examining results in relation to motor variability in the serve, several considerations should be mentioned. Firstly, results showed a negative relationship between the amount of variability in the movement of the hand holding the racket and performance in terms of accuracy and speed reached by the ball. Notwithstanding, an increase in the amount of variability in some kinematic variables is related to a decrease in the serve speed. On the other hand, complexity in hand trajectory would not be a predictor variable of neither accuracy nor ball speed reached in serves. Therefore, the analyzed movement is typified by the presence of fluctuations in the amount of variability analyzed. These fluctuations would appear as a response to serve performance demands relating to the accuracy and power requirements suggested in the task.

## Figures and Tables

**Table 1:** Dependent variables analyzed in the research. In each instant kinematic variable the standard deviation (SD) and the variation coefficient (CV) was analyzed

Variables measured in serve Instant (linear) kinematic	Non-linear
Movement duration of the hand holding the racket (dur)	Sample Entropy (SampEn) of hand position through X, Y, Z axes (posamx, posamy, posamz)
Initial and final position of the hand in X, Y, Z axes (poix, poiy, poiz; pofx, pofy, pofz)	
Distance travelled by the hand in X, Y, Z axes (dix, diy, diz)	
Maximum height reached by the hand in Z axis (piz)	SampEn of hand speed through X, Y, Z axes (vesamx, vesamy, vesamz)
Time reached in maximum height of the hand in Z axis (tpiz)	
Linear, final and total speed and acceleration of the hand in X, Y, Z axes (vlfx, vlfy, vlfz; vltx, vlty, vltz; alfx, alfy, alfz; altx, alty, altz)	
Maximum linear speed and acceleration of the hand in X, Y, Z axes (pvlx, pvly, pvlz; palx, paly, palz)	SampEn of hand acceleration through X, Y, Z axes (acsamx, acsamy, acsamz)
Recorded time in maximum linear speed and acceleration of the hand in X, Y, Z axes (tpvlx, tpvly, tpvlz; tpalx, tpaly, tpalz)	

**Table 2:** Significant correlation indexes between radial error and kinematic variables of the study

Variables		*N*	*Corr.*	*p*
Variation coefficient of maximum linear speed of the hand in X, Y and Z axes	pvlx_CV	17	.497	.042
	pvly_CV	17	.515	.034
	pvlz_CV	17	.522	.031

Standard deviation of maximum linear speed of the hand in Y and Z axes	pvly_SD	17	.492	.045
	pvlz_SD	17	.538	.026

Standard deviation of maximum speed of the hand in X axis	tpvlx_SD	17	.545	.024

Variation coefficient of maximum speed of the hand in Y axis	tpaly_CV	17	.501	.041

N=samples; Corr=correlation; p=significance; CV=variation coefficient; SD= standard deviation

**Table 3:** Significant correlation indexes between variable error and kinematic and non-linear variables

Variables		*N*	*Corr.*	*p*
Standard deviation in hand position at the beginning of the movement in Y axis	poiy_SD	17	−.486	.048

Variation coefficient of maximum speed of the hand in X axis	tpvlx_CV	17	.579	.015

Sample Entropy of hand position in the movement in X, Y and Z axes	posamx	17	.489	.046
	posamy	17	.483	.049
	posamz	17	.506	.038

Sample Entropy in linear speed of the hand in the movement in Y and Z axes	vesamy	17	.487	.047
	vesamz	17	.503	.039

Sample Entropy in linear acceleration of the hand in the movement in X axis	acsamx	17	.484	.049

N=samples; Corr=correlation; p=significance; CV=variation coefficient; SD= standard deviation

**Table 4:** Significant correlation indexes between ball speed and kinematic variables

Variables		*N*	*Corr.*	*p*
Standard deviation of hand position at the end of the movement in Y and Z axes	pofy_SD	17	−.577	.015
	pofz_SD	17	−.731	.001

Variation coefficient of hand position and the end of the movement in Y axis	pofy_CV	17	−.539	.026

Standard variation of the distance travelled by the hand in Y and Z axes	diy_SD	17	−.560	.019
	diz_SD	17	−.577	.015

Variation coefficient of the distance travelled by the hand in Y axis	diy_CV	17	−.492	.045

Variation coefficient of hand final speed in X axis	vlfx_CV	17	−.660	.004

Standard deviation of maximum linear acceleration of the hand in X and Z axes	tpalx_SD	17	−.576	.016
	tpalz_SD	17	−.572	.017

Variation coefficient of maximum linear acceleration of the hand in X axis	tpalx_CV	17	−.514	.035

N=samples; Corr=correlation; p=significance; CV=variation coefficient; SD= standard deviation
